# Supplementary material for: Comprehensive characterization of IFNγ signaling in acute myeloid leukemia reveals prognostic and therapeutic strategies
Source: Nat Commun. 2024 Feb 28;15:1821. doi: 10.1038/s41467-024-45916-6 (PMC10902356; doi:10.1038/s41467-024-45916-6)
Supplement: Supplementary file 1 — Supplementary information [file 41467_2024_45916_MOESM1_ESM.pdf]

Comprehensive Characterization of IFNγ Signaling in Acute Myeloid Leukemia Reveals Prognostic and Therapeutic Strategies

Supplemental Figure 1: Hallmark IFNγ signaling correlates with distinct cell types and T cell functional states in the AML microenvironment.
A. Distribution of Hallmark IFNγ response score across AML patients in bulk RNA profiling datasets. B. Correlation of cell types identified using CIBERSORTx immune deconvolution of bulk RNA profiling data and Hallmark IFNγ response. C. Correlation of hallmark IFNγ response with HLA class 1, D. HLA class 2, E. T-cell dysfunction score, F. T-cell exhaustion score, and G. T-cell senescence score. For all correlation figures, error band represents 95% confidence interval. T test was used to evaluate the significance of Pearson correlation.

Supplemental Figure 2: Quality control metrics of scRNA profiling across all cell types identified.
A. RNA counts by patients for each cell type included in the scRNA profiling cohort. B. Total number of RNA molecules detected within each cell type. C. Number of genes detected in each cell type. D. Fraction of cell reads originating from the mitochondrial genes across cell types. E. Fraction of RNA reads that mapped to the ribosomal genes. F. S phase score across cell types. G. G2M phase score across cell types. H. Percentage of major cell types identified among all patients profiled with scRNA profiling.

Supplemental Figure 3: Quality control metrics of scRNA profiling within AML blasts
A. Number of AML cells, B. Total number of RNA, C. The number of genes detected, D. Fraction of cell reads originating from the mitochondrial genes, E. Fraction of RNA reads that map to the ribosomal genes, F. S phase score, G. G2M phase score, and H. Fraction of cells represented by individual patients in each relevant phenotypic and cytogenetic group.

Supplemental Figure 4: IFNγ signaling is more prominent in monocytic and del7/7q AML with specific transcriptional regulation.
A. Histogram of Reactome IFNγ signaling across AML types. B. Independent validation of single cell IFNγ signaling score in diploid non-monocytic and diploid monocytic AML cells from ref 40. Center line represents the median and lower and upper bounds of box correspond to the first and third quartiles. Two-sided Wilcoxon test was used. C. Additional independent validation of single-cell IFNγ signaling score in diploid non-monocytic and diploid monocytic AML cells from ref 41. Center line represents the median and lower and upper bounds of box correspond to the first and third quartiles. Two-sided Wilcoxon test was used. D. Violin plot of Hallmark IFNγ response score by deconvoluted cell states. E. Interferon regulator factors (IRF) expression across AML types determined by SCENIC. F. Example flow cytometry gating strategy to identify AML blast cells. G. Representative histogram of pan-HLA class 2 (HLA-DR, -DQ, -DP) expression within AML blast cells as detected by flow cytometry and H. Quantification of MFI of pan-HLA class 2 (HLA-DR, -DQ, -DP) expression in diploid non-monocytic, del7/7q, and diploid monocytic AML patients tested.

Supplemental Figure 5: IFNγ interactions between CD8 T cells and NK cells is a prominent feature of diploid monocytic AML.
A. *IFNG* RNA expression in AML, CD4 T, CD8 T, and NK cells. B. Proportion of major cell types represented in different AML groups. C. Proportion of CD8 T-cell clusters represented in different AML groups (see Supplemental Figure 5E). D. Proportion of NK cell clusters represented in different AML groups (see Supplemental Figure 5F). E. UMAP projection of CD8

T cell clusters and cluster identities. F. UMAP projection of NK-cell clusters and cluster identities. G. Circos plot of the top predicted non-AML-to-AML ligand-receptor interactions within the diploid non-monocytic subset, H. The del5/5q subset, I. The del7/7q subset, and J. The double deletion (del7/7q and del5/5q) subset among the top 100 ligand-receptor interactions predicted by MultiNicheNet.

Supplemental Figure 6: *IFITM3* is independently prognostic for overall survival in newly diagnosed AML patients and its loss leads to AML cell death.
A. Spline of continuous log hazard ratio for overall survival by continuous RNA expression of *IFITM3* in the TCGA, B. MDACC, C. BEAT-AML1, and D. BEAT-AML2 datasets. E. Multivariable adjusted Cox regression model for overall survival among the patients in the combined bulk RNA profiling cohort adjusting for *IFITM3* expression, age, blast percentage, and cytogenetics. F. Unsupervised clustering of Hallmark IFNγ response signature genes and their effect on AML cell line fitness after CRISPR knockout from DepMap. G. Volcano plot showing 7 genes from Hallmark IFNγ response signature genes which had a significant negative effect on AML cell line viability after CRISPR deletion.

Supplemental Figure 7: Hallmark IFNγ response signature is not prognostic but the parsimonious IFNγ score is among newly diagnosed AML patients.
A. Correlation of *IFITM3* expression and venetoclax resistance in BEAT-AML data. B. Kaplan-Meier survival curve of the AML patients from TCGA, BeatAML and MDACC by median Hallmark IFNγ response signature split in bulk RNA profiling cohort. C. Schematic of development of parsimonious IFNγ score. D. Correlation of HLA class 1 expression and parsimonious IFNγ score. E. Correlation of HLA class 2 expression and parsimonious IFNγ score. F. Spline of continuous log hazard ratio for overall survival by the continuous parsimonious IFNγ score in the combined bulk RNA profiling datasets. G. Kaplan-Meier survival curve of the AML patients from Malani et al. by median parsimonious IFNγ score.

Supplemental Tables
Table S1: Patient and sample characteristics in single cell
Table S2: Cell type percentage by patient and cytogenetic groups
Table S3: List of antibodies used for clinical flow cytometry
Table S4: List of antibodies used for spectral flow cytometry
Table S5: List of antibodies used for Lunaphore multiplex IF
Table S6: Coefficient of IFNγ signatures fitted in the LASSO regression model

Figure S1

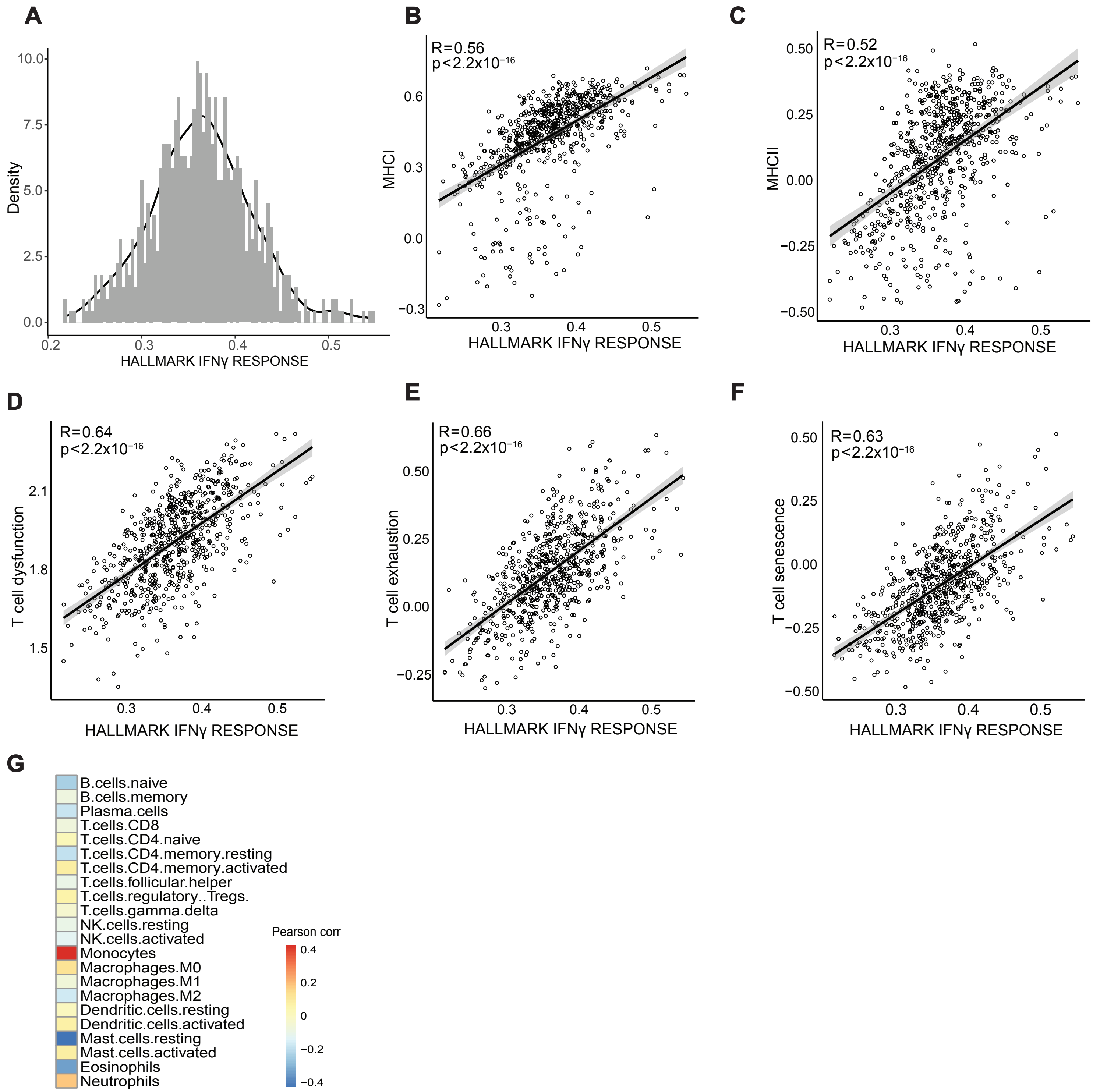
A
B
C
D
E
F
G
Density
HALLMARK IFNγ RESPONSE
R=0.56
p<2.2x10^-16
MHCI
R=0.52
p<2.2x10^-16
MHCII
R=0.64
p<2.2x10^-16
T cell dysfunction
R=0.66
p<2.2x10^-16
T cell exhaustion
R=0.63
p<2.2x10^-16
T cell senescence
B.cells.naive
B.cells.memory
Plasma.cells
T.cells.CD8
T.cells.CD4.naive
T.cells.CD4.memory.resting
T.cells.CD4.memory.activated
T.cells.follicular.helper
T.cells.regulatory..Tregs.
T.cells.gamma.delta
NK.cells.resting
NK.cells.activated
Monocytes
Macrophages.M0
Macrophages.M1
Macrophages.M2
Dendritic.cells.resting
Dendritic.cells.activated
Mast.cells.resting
Mast.cells.activated
Eosinophils
Neutrophils
Pearson corr

Figure S2

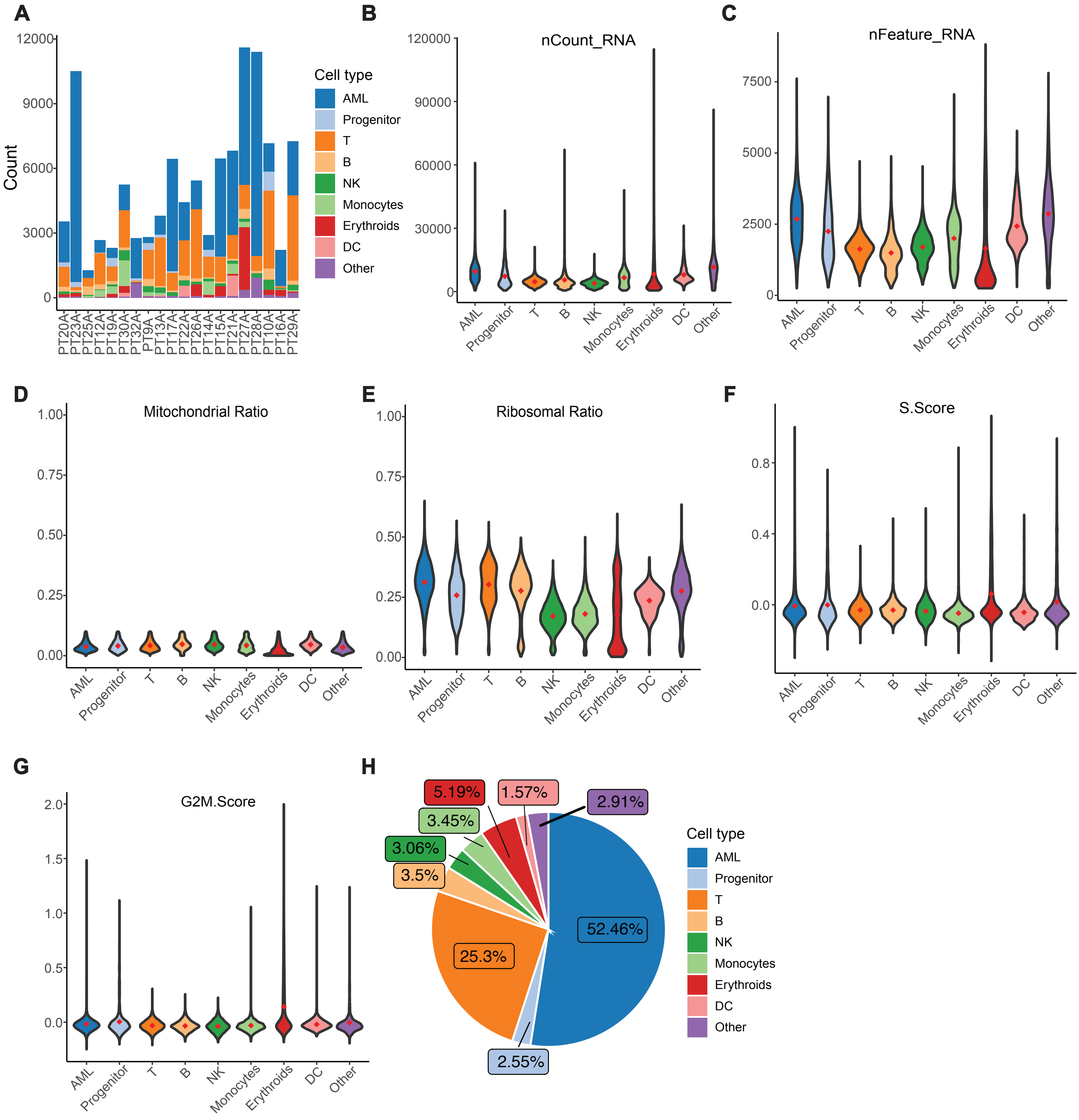
A
Count
Cell type
AML
Progenitor
T
B
NK
Monocytes
Erythroids
DC
Other
PT20A
PT23A
PT25A
PT12A
PT19A
PT30A
PT32A
PT9A
PT13A
PT17A
PT22A
PT26A
PT14A
PT15A
PT21A
PT27A
PT28A
PT10A
PT16A
PT29A
B
nCount_RNA
C
nFeature_RNA
D
Mitochondrial Ratio
E
Ribosomal Ratio
F
S.Score
G
G2M.Score
H
5.19%
1.57%
2.91%
3.45%
3.06%
3.5%
52.46%
25.3%
2.55%
Cell type
AML
Progenitor
T
B
NK
Monocytes
Erythroids
DC
Other

Figure S3

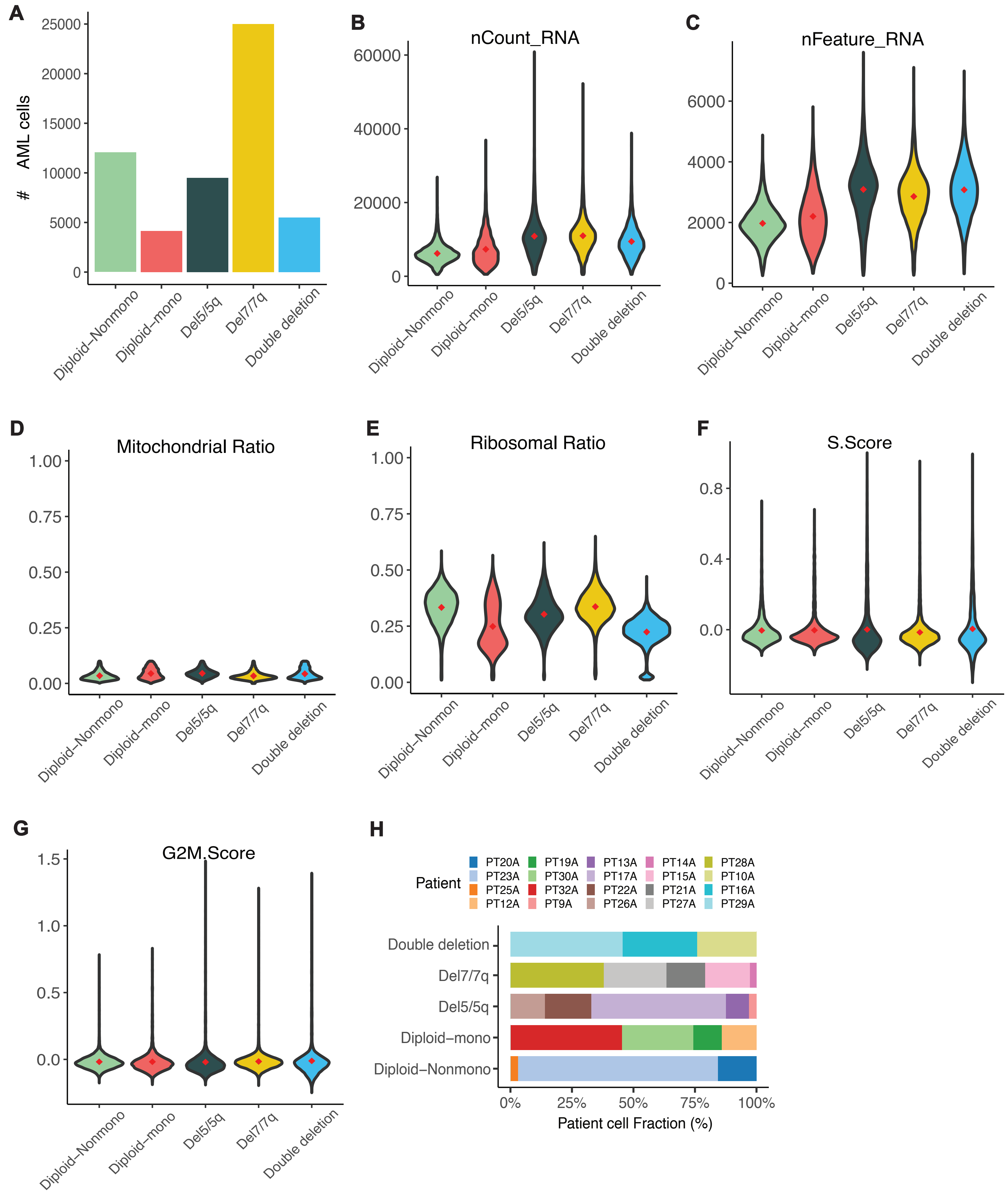
A
AML cells
25000
20000
15000
10000
5000
0
Diploid–Nonmono
Diploid–mono
Del5/5q
Del7/7q
Double deletion
B
nCount_RNA
60000
40000
20000
0
C
nFeature_RNA
6000
4000
2000
0
D
Mitochondrial Ratio
1.00
0.75
0.50
0.25
0.00
E
Ribosomal Ratio
Diploid–Nonmon
F
S.Score
0.8
0.4
0.0
G
G2M.Score
1.5
1.0
0.5
0.0
H
Patient
PT20A
PT23A
PT25A
PT12A
PT19A
PT30A
PT32A
PT9A
PT13A
PT17A
PT22A
PT26A
PT14A
PT15A
PT21A
PT27A
PT28A
PT10A
PT16A
PT29A
Double deletion
Del7/7q
Del5/5q
Diploid–mono
Diploid–Nonmono
0%
25%
50%
75%
100%
Patient cell Fraction (%)

Figure S4

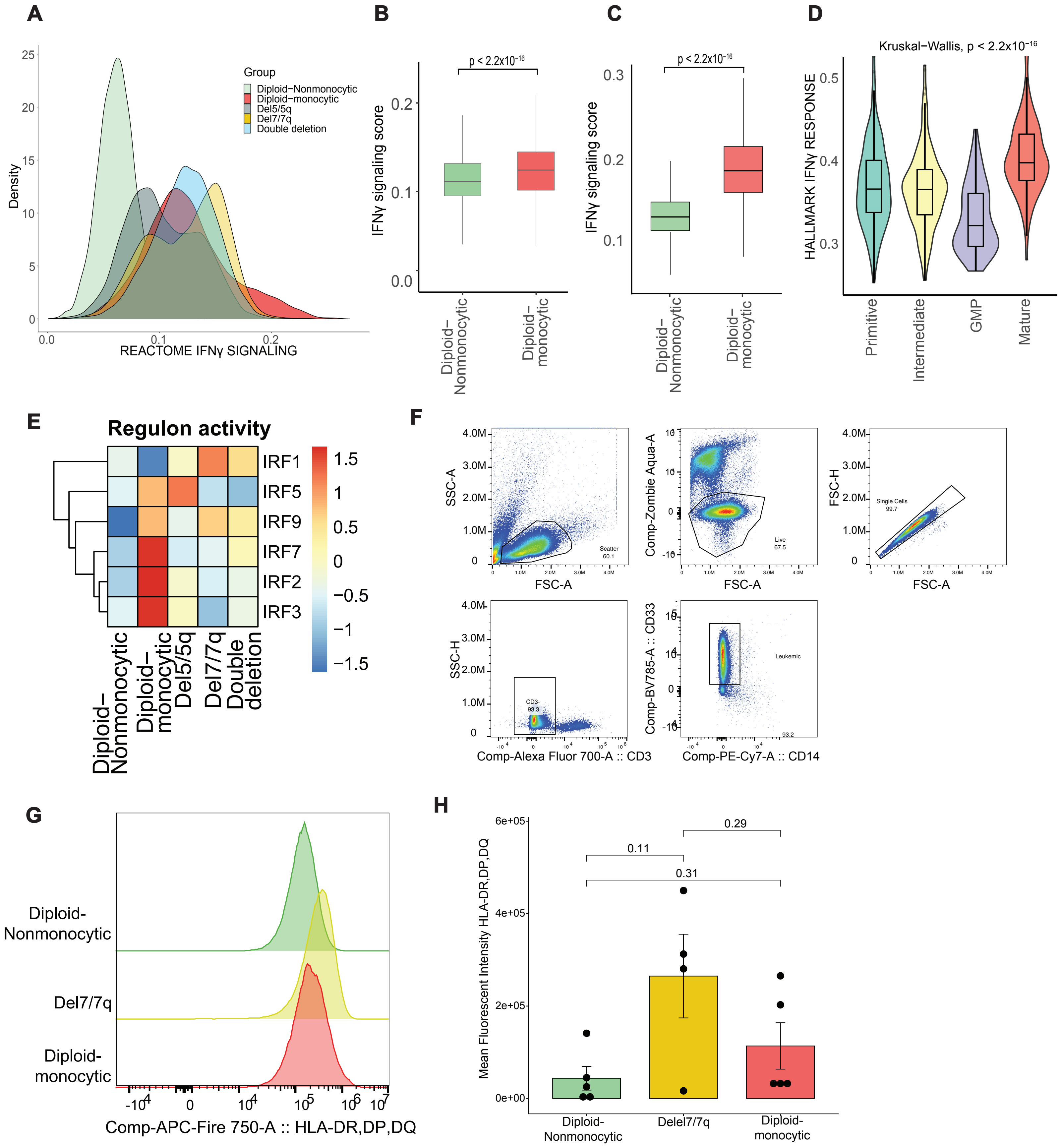

A
B
C
D
Group
Diploid−Nonmonocytic
Diploid−monocytic
Del5/5q
Del7/7q
Double deletion
Density
REACTOME IFNγ SIGNALING
p < 2.2x10^-16
IFNγ signaling score
Diploid−Nonmonocytic
Diploid−monocytic
Kruskal−Wallis, p < 2.2x10^-16
HALLMARK IFNγ RESPONSE
Primitive
Intermediate
GMP
Mature
E
Regulon activity
IRF1
IRF5
IRF9
IRF7
IRF2
IRF3
Diploid−Nonmonocytic
Diploid−monocytic
Del5/5q
Del7/7q
Double deletion
F
SSC-A
FSC-A
Scatter 60.1
Comp-Zombie Aqua-A
Live 67.5
FSC-H
Single Cells 99.7
SSC-H
CD3- 93.3
Comp-Alexa Fluor 700-A :: CD3
Comp-BV785-A :: CD33
Leukemic
93.2
Comp-PE-Cy7-A :: CD14
G
Diploid-Nonmonocytic
Del7/7q
Diploid-monocytic
Comp-APC-Fire 750-A :: HLA-DR,DP,DQ
H
Mean Fluorescent Intensity HLA-DR,DP,DQ
0.29
0.11
0.31
Diploid-Nonmonocytic
Delel7/7q
Diploid-monocytic

Figure S5

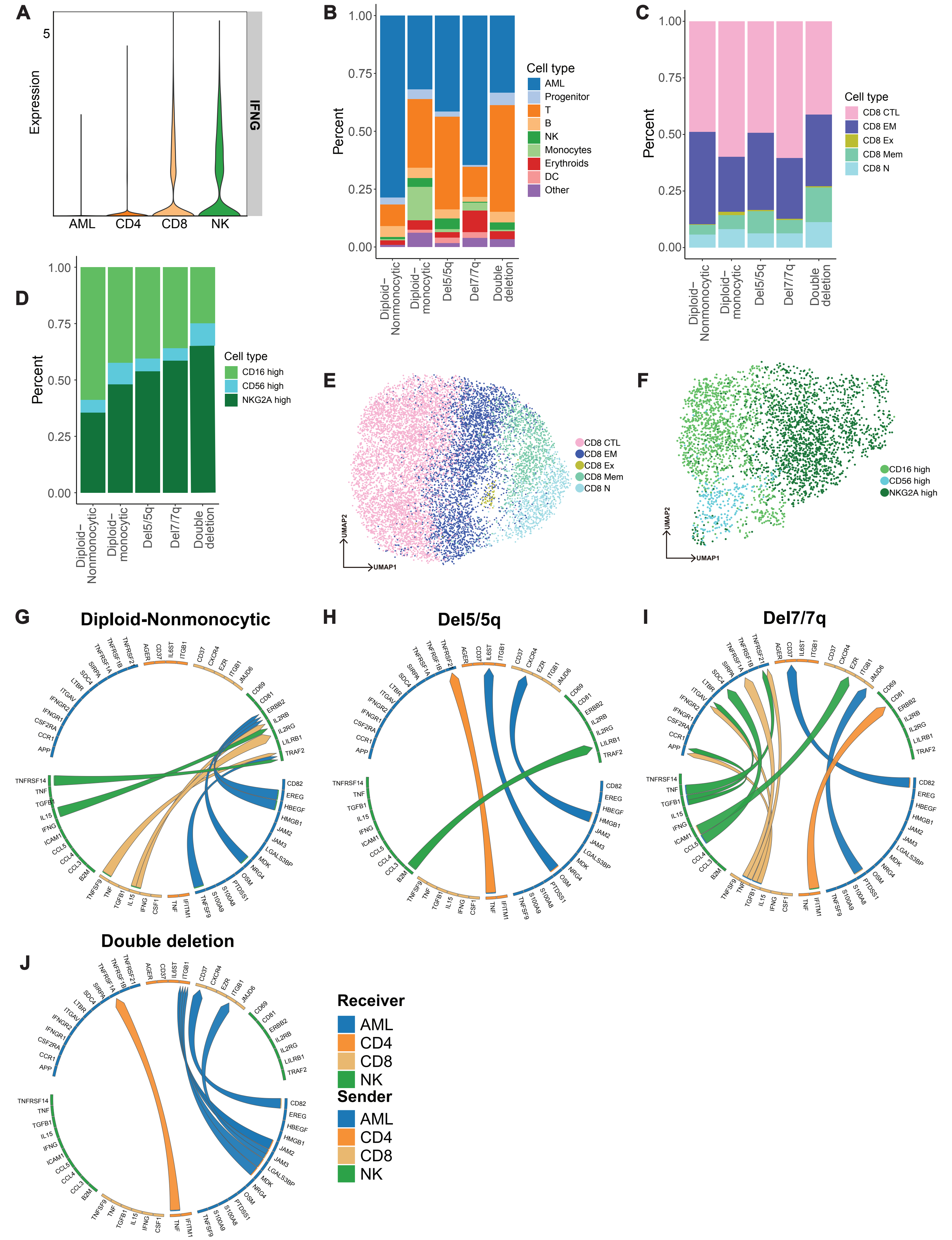

A
Expression
AML
CD4
CD8
NK
IFNG
B
Percent
Cell type
AML
Progenitor
T
B
NK
Monocytes
Erythroids
DC
Other
Diploid–Nonmonocytic
Diploid–monocytic
Del5/5q
Del7/7q
Double deletion
C
Cell type
CD8 CTL
CD8 EM
CD8 Ex
CD8 Mem
CD8 N
D
Cell type
CD16 high
CD56 high
NKG2A high
E
UMAP1
UMAP2
F
G
Diploid-Nonmonocytic
H
Del5/5q
I
Del7/7q
J
Double deletion
Receiver
AML
CD4
CD8
NK
Sender
AML
CD4
CD8
NK

Figure S6

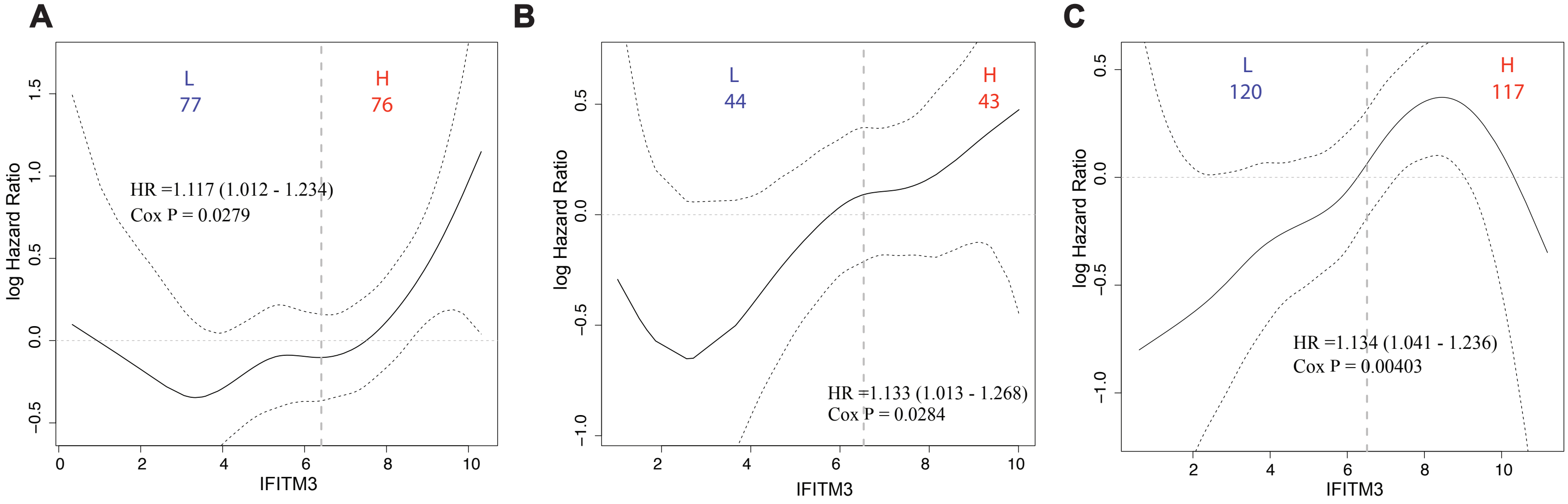

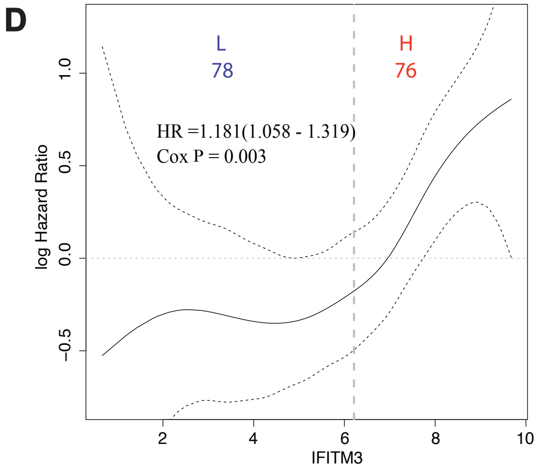

E

Variable	Hazard Ratio (95% CI)	p-value
Blast %	1.00 (1.00 - 1.01)	0.286
High IFITM3 (Above median)	1.38 (1.03 - 1.86)	0.033
Age >60 vs. Age <= 60	1.70 (1.30 - 2.22)	$9.15x10^{-5}$
Cytogenetic risk: Intermediate	3.21 (1.61 - 6.39)	$9.34x10^{-4}$
Cytogenetic risk: Unfavorable	3.86 (1.92 - 7.76)	$1.44x10^{-4}$

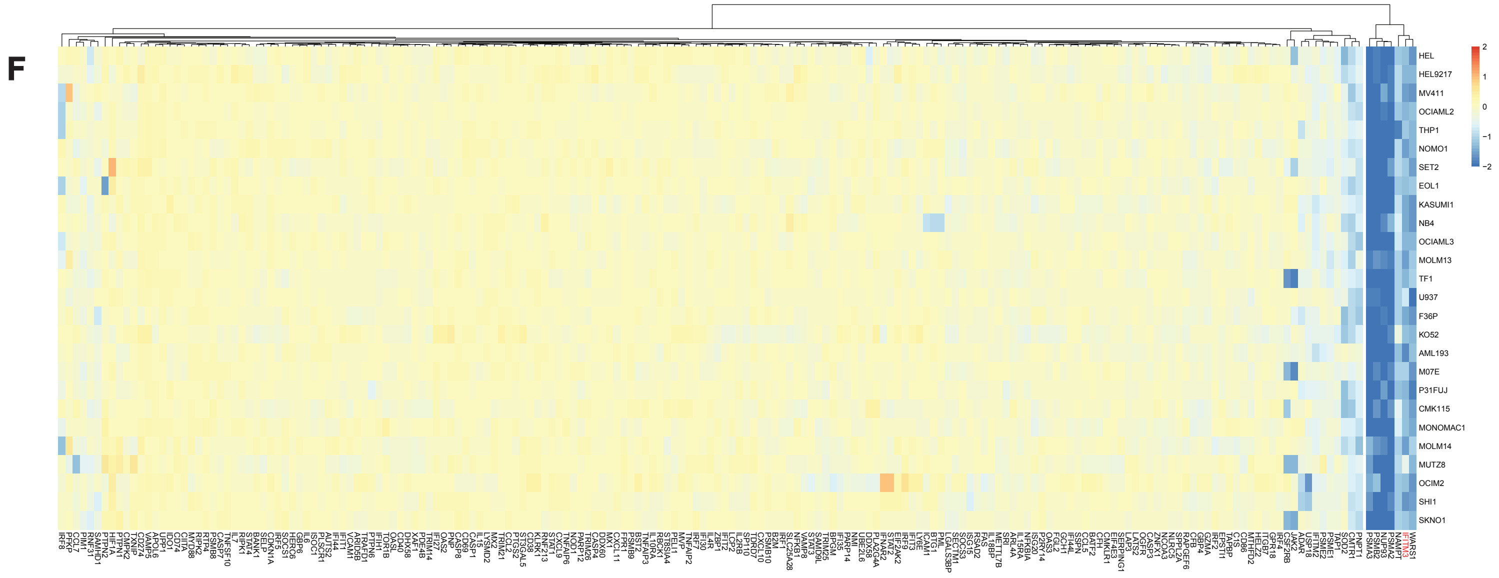

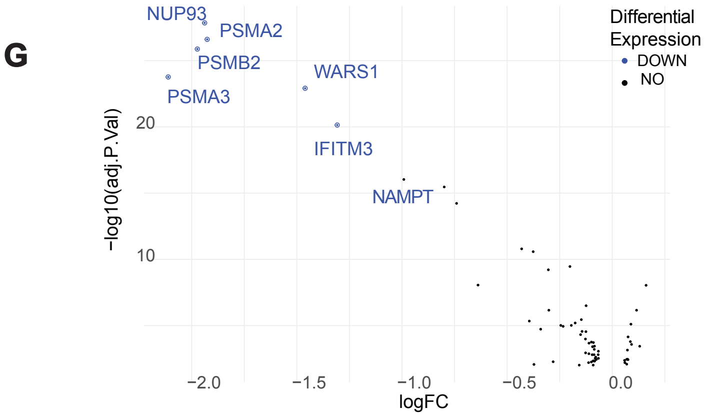

Figure S7

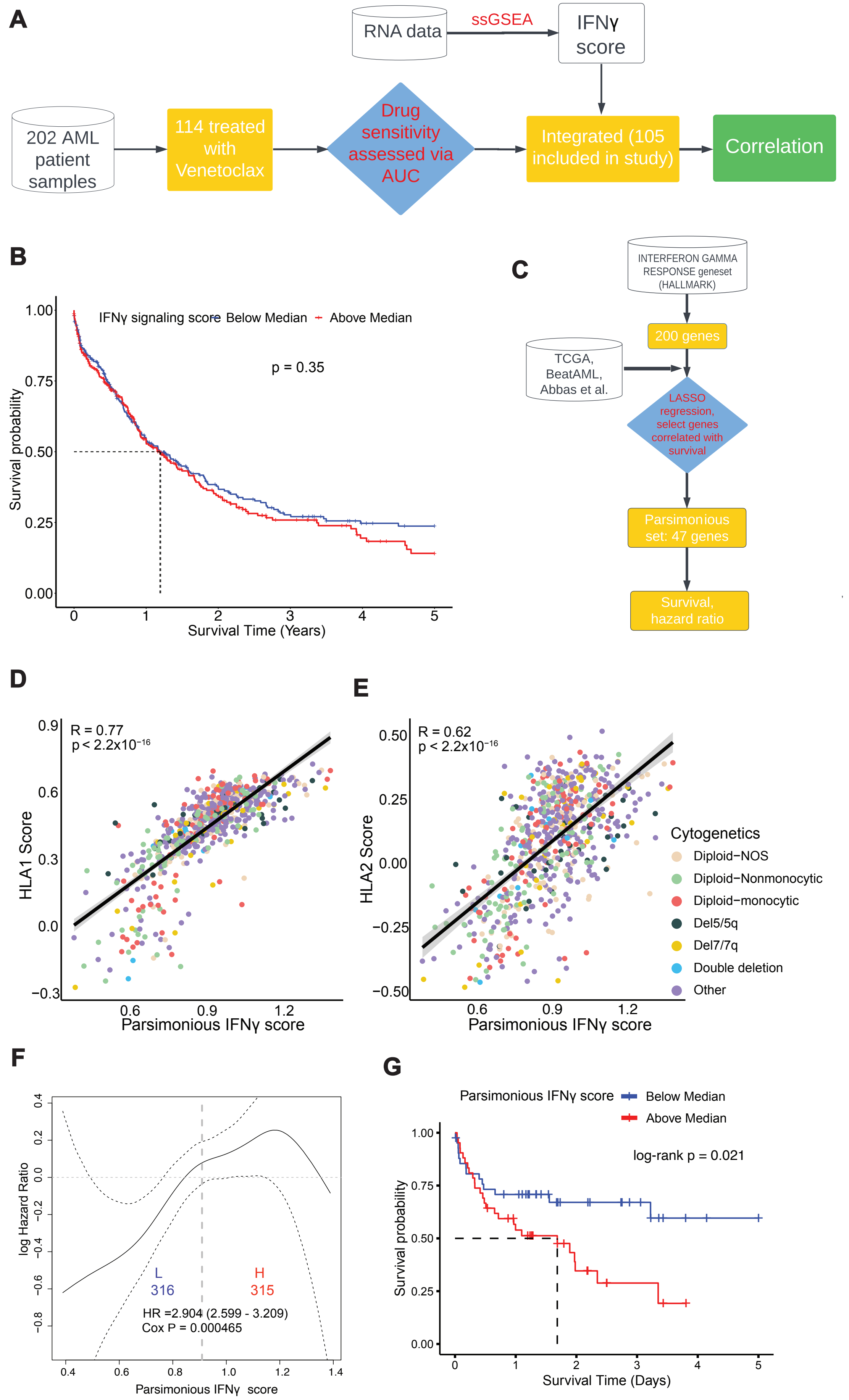

A
RNA data
ssGSEA
IFNγ score
202 AML patient samples
114 treated with Venetoclax
Drug sensitivity assessed via AUC
Integrated (105 included in study)
Correlation
B
IFNγ signaling score Below Median Above Median
p = 0.35
Survival probability
Survival Time (Years)
C
INTERFERON GAMMA RESPONSE geneset (HALLMARK)
200 genes
TCGA, BeatAML, Abbas et al.
LASSO regression, select genes correlated with survival
Parsimonious set: 47 genes
Survival, hazard ratio
D
R = 0.77
p < 2.2x10^-16
HLA1 Score
Parsimonious IFNγ score
E
R = 0.62
p < 2.2x10^-16
HLA2 Score
Parsimonious IFNγ score
Cytogenetics
Diploid−NOS
Diploid−Nonmonocytic
Diploid−monocytic
Del5/5q
Del7/7q
Double deletion
Other
F
log Hazard Ratio
L 316
H 315
HR =2.904 (2.599 - 3.209)
Cox P = 0.000465
Parsimonious IFNγ score
G
Parsimonious IFNγ score Below Median Above Median
log-rank p = 0.021
Survival probability
Survival Time (Days)

Supplementary tables for Wang and Reville *et al.*,

Comprehensive Characterization of IFNγ Signaling in Acute Myeloid Leuken Prognostic and Therapeutic Strategies

Contents

Table S1: Patient and sample characteristics in single cell
Table S2: Cell type percentage by patient and cytogenetic groups
Table S3: List of antibodies used for clinical flow cytometry
Table S4: List of antibodies used for spectral flow cytometry
Table S5: List of antibodies used for Lunaphore multiplex IF
Table S6: Coefficient of IFNγ signatures fitted in the LASSO regression model

Supplementary Table S1. Patient and sample characteristics in single cell

**Age**	**73 (52 - 87)**
**Female**	4/20 (20%)
**Race**	
White	16/20 (80%)
Asian	2/20 (10%)
Black	1/20 (5%)
Hispanic	1/20 (5%)
**AML Type**	
de novo	13/20 (65%)
Secondary	7/20 (35%)
**ELN2022 Risk**	
Favorable	4/20 (20%)
Intermediate	1/20 (5%)
Adverse	15/20 (75%)
**Cytogenetics**	
Diploid	7/20 (35%)
Complex	3/20 (15%)
Del7/7q	8/20 (40%)
Del5/5q	8/20 (40%)
Del17/17p	1/20 (5%)

Median (Range) or n/N (%)

Supplementary Table S2. Cell type percentage by patient and cytogenetic grou

Patient	AML	Progenitor	T	B	NK	Monocytes
PT9A	0.1041	0.1073	0.4773	0.1198	0.0956	0.0693
PT10A	0.1852	0.1213	0.5036	0.0747	0.0638	0
PT12A	0.2182	0.0116	0.5351	0.0945	0.0105	0.1173
PT13A	0.2326	0.0368	0.5869	0.0181	0.0652	0.0279
PT14A	0.2358	0.1103	0.3289	0.021	0.0379	0.2206
PT15A	0.7047	0.0026	0.1768	0.0124	0.0184	0
PT16A	0.7561	0.0104	0.0317	0	0.0172	0.0204
PT17A	0.8087	0.0129	0.1249	0.013	0.028	0
PT19A	0.2055	0.1688	0.2858	0.0751	0.0358	0.1528
PT20A	0.5361	0.0589	0.2604	0.0628	0.0331	0
PT21A	0.5731	0	0.1603	0.0166	0.0189	0.067
PT22A	0.4026	0	0.3654	0.0514	0.0566	0
PT23A	0.9309	0.0227	0.0126	0.0093	0.0043	0
PT25A	0.2853	0	0.3056	0.3041	0.0368	0.0525
PT26A	0.2449	0	0.5874	0.0305	0.023	0
PT27A	0.5491	0	0.0959	0.0389	0.0115	0.0221
PT28A	0.8311	0	0.0596	0.0109	0.0166	0
PT29A	0.3459	0	0.5442	0.0285	0.0327	0.0077
PT30A	0.2278	0	0.3288	0.024	0.0874	0.2297
PT32A	0.677	0.038	0.0105	0.0119	0.0036	0

Cytogenetic g	AML	Progenitor	T	B	NK	Monocytes
Diploid-Nonn	0.786	0.0291	0.0941	0.0462	0.0136	0.0044
Diploid-mono	0.3174	0.0405	0.2959	0.0451	0.0446	0.1441
Del5/5q	0.4144	0.0229	0.401	0.0386	0.0468	0.0131
Del7/7q	0.6378	0.0086	0.1271	0.0212	0.0174	0.0346
Double deleti	0.3312	0.0536	0.4586	0.0446	0.044	0.0061

ıps

Erythroids	DC	Other
0.0082	0	0.0185
0.0331	0	0.0184
0.0056	0	0.0071
0	0.0171	0.0155
0.0407	0	0.0048
0.0745	0	0.0105
0.1258	0	0.0385
0	0	0.0126
0.0678	0	0.0082
0.0371	0	0.0116
0.012	0.14	0.0122
0	0.1105	0.0135
0.0147	0	0.0056
0.0125	0	0.0031
0.0992	0	0.0149
0.2509	0	0.0315
0	0	0.0818
0.0092	0	0.0317
0.0589	0.0333	0.0101
0.0134	0	0.2456

Erythroids	DC	Other
0.0197	NA	0.0068
0.0398	0.0135	0.0592
0.0245	0.0242	0.0145
0.0917	0.0244	0.0373
0.035	0	0.0269

Table S3: List of antibodies used for clinical flow cytometry

Target	Company	Clone	Catalog No.	Dilution
CD34	BD	8G12	348057	1:50
CD33	BD	P67.2P67.6	340474	1:50
CD64	BD	10.1	558592	1:50

Table S4: List of antibodies used for spectral flow cytometry

Target	Conjugation	Company	Clone	Catalog No.	Volume/Dilution
CD3	Alexa Fluor 700	BioLegend	HIT3a	300324	2.5
CD33	Brilliant Violet 785	BioLegend	WM53	303427	2.5
CD14	PE/Cyanine7	BioLegend	M5E2	301814	2.5
CD64	PerCP/Cyanine 5.5	BioLegend	10.1	305024	2.5
HLA-E	Brilliant Violet 421	BioLegend	3D12	342612	2.5
CD34	PE Dazzle 594	BioLegend	581	343534	2.5
HLA-DR,DP, DQ	APC Fire 750	BioLegend	Tü39	361712	2.5
Live/Dead	Zombie Aqua	BioLegend		77143	1 in 1000

Table S5: List of antibodies used for Lunaphore multiplex IF

Target	Host	Clone	Mfc	Dilution	Cat No.	Reactivity
CD34	Rb	EP373Y	Abcam	1:50	Ab81289	Ms, Rt, Hu
HLAE	Ms	1A4G3	ProteinTech	0.111111111	66530-1-Ig	Hu
CD3	Ms	3F3A1	ProteinTech	0.111111111	60181-1-Ig	Hu
CD45	Rb	BL-178-12C7	Bethyl	0.215277778	A700-012	Hu
IFITM3	Rb	D8E8G	Cell Signaling	1:50	59212	Hu

Supplementary Table S6. Coefficient of IFNγ signatures fitted in the LASSO regr

Note: Genes with non-zero coefficient w

Gene	Coefficient
STAT1	-0.0003937
IFI35	-0.0014535
IRF1	-0.0013863
IRF9	0.00089371
OASL	-0.0027547
PSMB8	0.01669831
IFITM3	0.00055714
DDX60	-0.031288
IFI27	0.0001721
LY6E	1.77E-06
PLA2G4A	0.04636913
NLRC5	-0.0088095
PTPN2	0.04832014
SOCS1	0.00155595
ISG20	0.00017374
PARP12	-0.0030237
TRIM26	-0.0114742
GPR18	-0.0157695
CCL5	0.00213994
CFB	-0.0064625
TXNIP	1.16E-05
PNP	0.00022083
CASP7	0.0236313
HLA-G	0.02043786
MT2A	-6.26E-05
UPP1	0.00056413
TDRD7	-0.0076651
VAMP8	-0.0020368
CD38	-0.0006928
ZBP1	-5.01E-05
MVP	-0.0001587
IL7	0.02041031
B2M	-1.96E-05
CD69	-0.0001847
CFH	0.00720246
TAPBP	0.00334266
SSPN	-0.0847873
LATS2	0.00663012
SLAMF7	-0.0001231
ISOC1	-0.023754
GZMA	0.00320683
CD74	-5.69E-05
CASP4	-0.0030153

C1R	-0.0125684
CMKLR1	0.02774187
VAMP5	0.00358027
ARID5B	0.01314079

ession model

ʋere retained to form the parsimonious IFNγ signature
