# Supplementary material for: Comprehensive characterization of IFNγ signaling in acute myeloid leukemia reveals prognostic and therapeutic strategies
Source: Nat Commun. 2024 Feb 28;15:1821. doi: 10.1038/s41467-024-45916-6 (PMC10902356; doi:10.1038/s41467-024-45916-6)
Supplement: Supplementary file 3 — Description of Additional Supplementary Files [file 41467_2024_45916_MOESM3_ESM.pdf]

<u>Description of Additional Supplementary Files</u>

Supplementary Data Legends

Supplementary Data1: Patient characteristics in bulk RNA cohort

Supplementary Data 2: Correlation of GO, Reactome, Hallmark pathway scores with IFNγ signaling score

Supplementary Data 3: Top 100 differentially expressed genes for all cell types included in this study

Supplementary Data 4: Top 100 predicted ligand-receptor interactions across all cytogenetic groups

Supplementary Data 5: Top 50 predicted ligand-receptor interactions within Diploid-monocytic only

Supplementary Data 6: Correlations of IFNγ signaling score with all genes in AML cells
