# Supplementary material for: Comprehensive characterization of IFNγ signaling in acute myeloid leukemia reveals prognostic and therapeutic strategies
Source: Nat Commun. 2024 Feb 28;15:1821. doi: 10.1038/s41467-024-45916-6 (PMC10902356; doi:10.1038/s41467-024-45916-6)
Supplement: Supplementary file 5 — Reporting Summary [file 41467_2024_45916_MOESM5_ESM.pdf]

nature portfolio

Corresponding author(s): Hussein A. Abbas

Last updated by author(s): Dec 17, 2023

Reporting Summary

Nature Portfolio wishes to improve the reproducibility of the work that we publish. This form provides structure for consistency and transparency in reporting. For further information on Nature Portfolio policies, see our Editorial Policies and the Editorial Policy Checklist.

Statistics

For all statistical analyses, confirm that the following items are present in the figure legend, table legend, main text, or Methods section.

n/a	Confirmed	
☐	☒	The exact sample size (*n*) for each experimental group/condition, given as a discrete number and unit of measurement
☐	☒	A statement on whether measurements were taken from distinct samples or whether the same sample was measured repeatedly
☐	☒	The statistical test(s) used AND whether they are one- or two-sided<br>*Only common tests should be described solely by name; describe more complex techniques in the Methods section.*
☐	☒	A description of all covariates tested
☐	☒	A description of any assumptions or corrections, such as tests of normality and adjustment for multiple comparisons
☐	☒	A full description of the statistical parameters including central tendency (e.g. means) or other basic estimates (e.g. regression coefficient) AND variation (e.g. standard deviation) or associated estimates of uncertainty (e.g. confidence intervals)
☐	☒	For null hypothesis testing, the test statistic (e.g. *F*, *t*, *r*) with confidence intervals, effect sizes, degrees of freedom and *P* value noted<br>*Give P values as exact values whenever suitable.*
☒	☐	For Bayesian analysis, information on the choice of priors and Markov chain Monte Carlo settings
☒	☐	For hierarchical and complex designs, identification of the appropriate level for tests and full reporting of outcomes
☐	☒	Estimates of effect sizes (e.g. Cohen's *d*, Pearson's *r*), indicating how they were calculated

Our web collection on statistics for biologists contains articles on many of the points above.

Software and code

Policy information about availability of computer code

Data collection	Rv.4.2.1, Cell Ranger, InferCNV, GraphPad Prism10
Data analysis	All statistical analyses and figures were performed using Rv.4.2.1 and Prism10

For manuscripts utilizing custom algorithms or software that are central to the research but not yet described in published literature, software must be made available to editors and reviewers. We strongly encourage code deposition in a community repository (e.g. GitHub). See the Nature Portfolio guidelines for submitting code & software for further information.

Data

Policy information about availability of data

All manuscripts must include a data availability statement. This statement should provide the following information, where applicable:

- Accession codes, unique identifiers, or web links for publicly available datasets
- A description of any restrictions on data availability
- For clinical datasets or third party data, please ensure that the statement adheres to our policy

The single cell sequencing data generated in this study have been deposited in the GEO database with the accession number GSE239721. Source data are provided with this paper.

Research involving human participants, their data, or biological material

Policy information about studies with human participants or human data. See also policy information about sex, gender (identity/presentation), and sexual orientation and race, ethnicity and racism.

Reporting on sex and gender	The study was performed in adult (aged ≥18 years old) AML patients who are newly diagnosed and have bone marrow samples available for analysis from our Leukemia Sample Bank . Sex and gender were not considered in study design.
Reporting on race, ethnicity, or other socially relevant groupings	Not applicable
Population characteristics	New diagnosed AML patients,median age of 73 years (range 52-87)
Recruitment	New diagnosed AML patients were considered for study entry. Sample sizes were determined with the intent to capture a representative cohort of AML patients, encompassing the principal molecular subgroups identified in current research. This decision was also informed by practical considerations including budgetary constraints and the availability of samples within our databank. Due to the nature of our study, which necessitated the correlation of molecular findings with clinical outcomes, randomization and blinding were not employed.
Ethics oversight	A written informed consent that was approved by the internal review board of University of Texas M D Anderson Cancer Center was obtained. The study was conducted in accordance with the principles of Declaration of Helsinki.

Note that full information on the approval of the study protocol must also be provided in the manuscript.

Field-specific reporting

Please select the one below that is the best fit for your research. If you are not sure, read the appropriate sections before making your selection.

☒ Life sciences ☐ Behavioural & social sciences ☐ Ecological, evolutionary & environmental sciences

For a reference copy of the document with all sections, see nature.com/documents/nr-reporting-summary-flat.pdf

Life sciences study design

All studies must disclose on these points even when the disclosure is negative.

Sample size	20 patients for single cell study. Samples were selected based on cytogenetic abnormalities of patients. with the intent to capture a representative cohort of AML patients, encompassing the principal molecular subgroups identified in current research. This decision was also informed by practical considerations including budgetary constraints and the availability of samples within our databank.
Data exclusions	No specific data was excluded
Replication	T cell-AML co-culture experiment was performed triplicate on each sample and all attempts were successful. Multiplexing IF experiments were repeated 2 and 10 times each. All were successful.
Randomization	Not applicable. Newly diagnosed patients were selected based on cytogenetic abnormalities, and available bone marrows to be analyzed.
Blinding	Due to the nature of our study, which necessitated the correlation of molecular findings with clinical outcomes, blinding was not employed.

Reporting for specific materials, systems and methods

We require information from authors about some types of materials, experimental systems and methods used in many studies. Here, indicate whether each material, system or method listed is relevant to your study. If you are not sure if a list item applies to your research, read the appropriate section before selecting a response.

Materials & experimental systems

n/a	Involved in the study	
☐	☒	Antibodies
☐	☒	Eukaryotic cell lines
☒	☐	Palaeontology and archaeology
☒	☐	Animals and other organisms
☒	☐	Clinical data
☒	☐	Dual use research of concern
☒	☐	Plants

Methods

n/a	Involved in the study	
☒	☐	ChIP-seq
☐	☒	Flow cytometry
☒	☐	MRI-based neuroimaging

Antibodies

Antibodies used	CD3(BioLegend, HIT3a,300324), CD33(BioLegend,WM53, 303427), CD14(BioLegend,M5E2, 301814), CD64(BioLegend,10.1, 305024), HLA-E(BioLegend,3D12, 342612), CD34(BioLegend, 581, 343534), HLA-DR/DP/DQ (BioLegend,Tü39, 361712), IFITM3(Cell Signaling Technology, D8E8G, 59212), CD45(Bethyl, BL-178-12C7, A700-012); CD64(BD, 10.1, 558592)
Validation	All antibodies in the study were used according to the user manuals and validation statements can be found on the respective manufacture website (https://www.abcam.com/products/primary-antibodies/cd34-antibody-ep373y-ab81289.html; https://www.cellsignal.com/products/primary-antibodies/ifitm3-d8e8g-xp-rabbit-mab/59212; https://www.fortislife.com/products/primary-antibodies/rabbit-anti-cd3e-recombinant-monoclonal-antibody-bl-298-5d12/BETHYL-A700-016; https://www.ptglab.com/products/HLA-E-Antibody-66530-1-Ig.htm;https://www.biolegend.com/de-at/explore-new-products/pe-cyanine7-anti-human-cd14-antibody-2729?GroupID=BLG10263; https://www.biolegend.com/de-at/products/apc-fire-750-anti-human-hla-dr-dp-dq-antibody-16462; https://www.biolegend.com/de-at/products/percp-cyanine5-5-anti-human-cd64-antibody-8195)

Eukaryotic cell lines

Policy information about cell lines and Sex and Gender in Research

Cell line source(s)	THP1 cells (Male), and MOLM13(Male) cells from collaborators; Primary AML samples from 9 patients (6 male and 3 female)
Authentication	Cell line identities were confirmed by STR DNA Analysis.
Mycoplasma contamination	All cell lines tested negative for mycoplasma contamination.
Commonly misidentified lines (See ICLAC register)	No commonly misidentified cell lines were used in the study.

Plants

Seed stocks	*Report on the source of all seed stocks or other plant material used. If applicable, state the seed stock centre and catalogue number. If plant specimens were collected from the field, describe the collection location, date and sampling procedures.*
Novel plant genotypes	*Describe the methods by which all novel plant genotypes were produced. This includes those generated by transgenic approaches, gene editing, chemical/radiation-based mutagenesis and hybridization. For transgenic lines, describe the transformation method, the number of independent lines analyzed and the generation upon which experiments were performed. For gene-edited lines, describe the editor used, the endogenous sequence targeted for editing, the targeting guide RNA sequence (if applicable) and how the editor was applied.*
Authentication	*Describe any authentication procedures for each seed stock used or novel genotype generated. Describe any experiments used to assess the effect of a mutation and, where applicable, how potential secondary effects (e.g. second site T-DNA insertions, mosiacism, off-target gene editing) were examined.*

Flow Cytometry

Plots

Confirm that:

☒ The axis labels state the marker and fluorochrome used (e.g. CD4-FITC).

☒ The axis scales are clearly visible. Include numbers along axes only for bottom left plot of group (a 'group' is an analysis of identical markers).

☒ All plots are contour plots with outliers or pseudocolor plots.

☒ A numerical value for number of cells or percentage (with statistics) is provided.

Methodology

Sample preparation	Patient peripheral blood mononuclear cell samples that had been frozen in 90% FBS with 10% dimethyl sulfoxide were obtained from the Sample Bank
Instrument	Cytek Aurora 5-laser Spectral Flow Cytometer
Software	FlowJo
Cell population abundance	Unsorted primary AML patient samples
Gating strategy	We gated on FSC-med, SSC-low to identify the lymphoid/myeloid cell population based on intensity patterns. Using the Zombie Aqua stain, live cells were filtered for, based on the separation of the two populations by intensity pattern. Doublets were filtered by comparing the FSC-A/FSC-H population and removing those with increased area for a decreased height. Starting standard gates were identified using the healthy control population to estimate the “expected” expression and

fluorescence intensity, with minor changes based on intensity pattern differentiation. Gates for leukemic cells were done on the CD3- population to remove T cells, and none of the samples had CD3 expression in blasts based on diagnostic clinical flow cytometry data. Markers from clinical flow data were also used to identify leukic populations, specifically CD14, CD33, and CD34. Non-monocytic populations were CD64-. For markers in which two or more distinct populations could not be identified, only comparisons of mean fluorescence intensity versus the other groups were done.

☒ Tick this box to confirm that a figure exemplifying the gating strategy is provided in the Supplementary Information.
